# Adaptation and validation of the modified Egyptian Arabic version of Addenbrooke’s Cognitive Examination III (VI-ACE-III) for assessing cognitive impairment in visually impaired elderly

**DOI:** 10.1186/s12877-025-05784-1

**Published:** 2025-03-04

**Authors:** Samar Mamdouh Abdelsalam, Abeer Hassan Mohamed Matter, Hazem Mohamed El-Hariri, Ahmed Hassan Assaf, Mohamed Shawky Khater, Heba Mohamed Tawfik

**Affiliations:** 1 Faculty of Medicine, Ain Shams University, Cairo, Egypt; 2 National Research Centre, Cairo, Egypt

**Keywords:** Dementia, Addenbrooke's cognitive examination, Mild cognitive impairment, Vision impairment, VI-ACE-III, Arabic

## Abstract

**Background:**

Vision impairment affects the accuracy of cognitive test outcomes, emphasizing the need for developing cognitive screening tools designed for visually impaired individuals, especially considering global aging trends. This study aimed to develop a modified, validated version of the Vision-Impairment version of Addenbrooke's Cognitive Examination III (VI-ACE-III) for Arabic-speaking elderly individuals with vision impairment in Egypt. In addition, the study aimed to assess the accuracy of VI-ACE-III in diagnosing dementia and mild cognitive impairment (MCI).

**Methods:**

The VI-ACE-III was developed using large printed formats and verbal substitution of the vision-dependent items. One hundred and eighty participants aged ≥ 60, with varying degrees of vision impairment (including moderate, severe, and blindness), were divided into three equal groups: 60 individuals with MCI, 60 with mild to moderate dementia, and 60 with cognitively intact controls. Receiver operating characteristics (ROC) curves were plotted to assess the accuracy of the test screening.

**Results:**

ROC analysis for dementia established an optimal cut-off point of 84 out of 115, demonstrating 100.0% sensitivity, 98.3% specificity, and an area under the curve (AUC) of 0.983, based on the comparison between the dementia and MCI groups. The analysis for MCI determined an optimal cut-off point of 94 out of 115, with 95.0% sensitivity, 96.7% specificity, and an AUC of 0.983 compared to controls. The VI-ACE-III demonstrated significant Cronbach's alpha values (α = 0.866, α = 0.771), indicating strong internal consistency within the dementia and MCI groups.

**Conclusions:**

The VI-ACE-III showed good sensitivity and specificity for assessing dementia and MCI in Arabic-speaking elderly individuals with visual impairment (VI) in Egypt. Regular screening and interventions are crucial for managing and preventing the deterioration of cognitive dysfunction and vision impairment in the elderly population.

**Supplementary Information:**

The online version contains supplementary material available at 10.1186/s12877-025-05784-1.

## Introduction

The United Nations Population Division and World Population Prospects indicate that in 2021, individuals aged 60 and above comprised approximately 8.6% of Egypt's total population. This percentage is projected to increase to 14.6% by 2050 [1]. The prevalence of cognitive and vision impairment is anticipated to rise due to the ongoing global aging trend [2]. Cognitive dysfunction is associated with vision impairment, attributed to changes in physical functioning, heightened social isolation, and depression, which affect brain function and structure [3]. Additionally, increased processing effort contributes to a higher cognitive load required to perform tasks [4]. Visual function affects cognitive testing results, as these tests measure several cognitive domains dependent on visual skills [5]. This may negatively affect the assessment, resulting in an overestimation of cognitive impairment [6], as individuals with vision impairment may struggle to maintain equivalent focus during the test, resulting in varied performance outcomes [7]. Earlier attempts to tailor cognitive assessments for individuals with VI by removing vision-dependent items may result in an inaccurate evaluation of cognitive performance. This occurs if the deleted items are more or less challenging than the remaining items, thereby reducing sensitivity [8]. Moreover, deleting items can potentially decrease retest reliability [9]. Alternatively, visiondependent items have been suggested as spoken or tactile versions [10]. Addenbrooke's Cognitive Examination III (ACE-III) is a widely used screening test for cognitive function [11] that has been validated in Egyptian Arabic and has demonstrated a high degree of differentiation in diagnosing both dementia and MCI [12].

This study aimed to validate and assess the psychometric properties of an adapted Egyptian Arabic version of ACE-III for assessing cognitive impairment in visually impaired elderly individuals. We ensured that visually dependent items, presented in verbal or large printed formats, were of comparable difficulty and assessed the exact cognitive domains as the original test.

## Methods

### Participants

This study included 180 visually impaired participants aged ≥ 60 years recruited from the Ophthalmology Department and the Memory Clinic at Ain Shams University hospitals. Participants were selected based on the World Health Organization (WHO) criteria for visual impairment [13].

### Inclusion criteria

- Males and females aged ≥ 60 years
- Formal education

### Exclusion criteria

- Participants diagnosed with severe dementia or depression. Depression was assessed using the Geriatric Depression Scale (GDS), Arabic version, with a cut-off score of ≥ 5 indicating the presence of depressive symptoms [14].
- The Arabic version of the Cornell Scale for Depression in Dementia (CSDD) was utilized to evaluate depression in participants with dementia, with a score of ≥ 8 suggesting significant depressive symptoms [15].
- Participants with hearing impairments, as well as those with neurological or psychiatric conditions that may HINDER cognitive assessment.
- Participants requiring urgent sensory interventions that could affect their participation in the study.

### Procedure

**Fig. 1:**
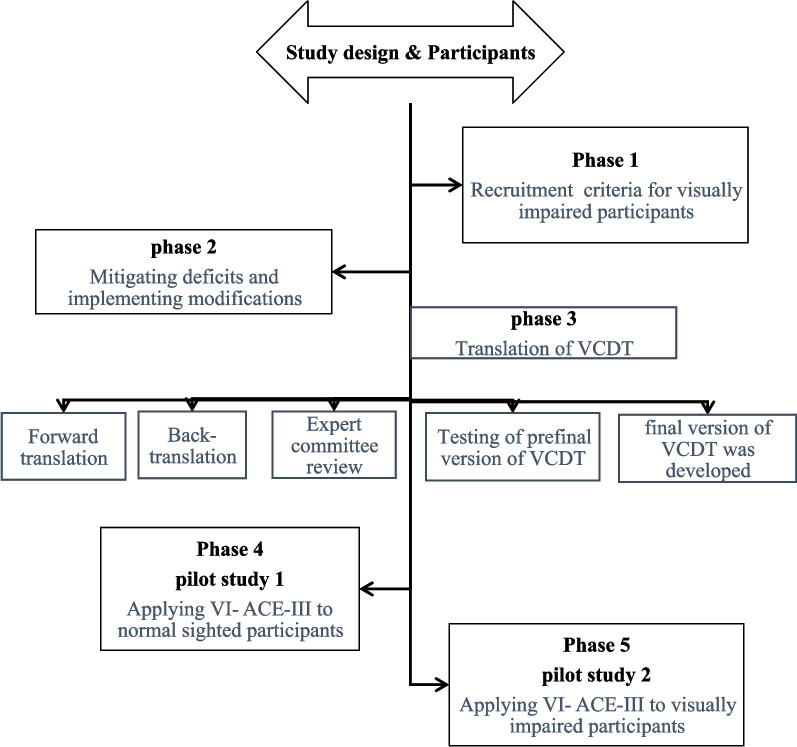
Flow diagram for participant recruitment: initial phases and overview of pilot study

The procedure involved multiple phases (Fig. 1) to comprehensively assess the impact of VI on cognitive performance, incorporating necessary modifications for different levels of vision loss. All phases were designed to maintain transparency and reproducibility, following the guidelines outlined by Zogmeister [16].

#### Phase 1

This phase aimed to determine the impact of different grades of VI on cognitive performance. Thirty participants with different levels of vision impairment underwent the standard version of the ACE-III Test. Participants in these categories are classified based on their best eye's visual acuity. VI was assessed following WHO criteria for visual impairment into moderate VI Visual acuity ranging from 6/18 to 6/60., severe VI Visual acuity ranging from 6/60 to 3/60.and blindness Visual acuity ranging from 3/60 to 1/60.exhibited more substantial declines in their ACE-III Test scores.

#### Phase 2

This phase focused on modifying cognitive assessments to accommodate varying degrees of vision impairment. Modifications included verbal substitutions and large-print formats, which improved test performance compared to tactile versions.

#### Phase 3

It ran parallel to the first two phases. The Verbal Clock Drawing Test (VCDT) [17] was translated after obtaining prior approval from the original developer, Professor Steven P. Cercy. The translation process included the following stages (as shown in Fig. 1): a. Forward translation conducted by a professional translator into Arabic. b. Back translation was performed by a different professional translator into English, who had no prior knowledge about the test. c. Review by an expert committee comprising specialists in medical research, health professionals, researchers engaged in translation studies, and one patient interested in health education. d. The pre-final version of the test was evaluated with 30 individuals aged ≥ 60 years. Responses to all items were reviewed by the expert committee, which included a modification to the first question regarding the clock face, where both 'circle' and 'square' received a score of 2, unlike in the original test. The final version of the translated Arabic VCDT was determined based on the results of preference testing.

#### Phase 4 (pilot study 1)

The modifications performed in Phase 2 were applied to 30 normal-sighted participants. The objective was to guarantee that these modifications evaluate the same cognitive domains.

Modifications to ACE III test (VI-ACE III test): In the language domain, the modifications entailed verbalizing two sentences instead of writing them. Object naming is largely printed with enhanced contrast and the five words to be read.

For the visuospatial domain, intersecting infinity loops and cube drawings were printed in a larger format with enhanced thickness and contrast. The clock drawing test was substituted with the Arabic version of VCDT. Counting dots and fragmented letters were printed in large format with enhanced contrast.

#### Phase 5 (pilot study 2)

The modified VI-ACE III was administered to 30 visually impaired participants to assess its effectiveness and ensure it adequately measured cognitive domains for individuals with varying degrees of VI.

### Design

A case–control study design was adopted from January 2023 to March 2024. It was conducted in two main phases: a preliminary study and a final study, each one had distinct objectives and processes.

#### Preliminary study

The main focus of this study was to develop and refine the cognitive assessments to ensure their suitability for individuals with varying degrees of visual impairment. This phase involved a pilot phase to test and modify the ACE-III and the Verbal Clock Drawing Test (VCDT). The preliminary study encompassed several activities, including phases 1 to 5, as demonstrated in Fig. 1.

#### Final study

This study aimed to assess cognitive performance, comparing scores across different levels of vision impairment and sitting scores to diagnose dementia and MCI. VI-ACE III was applied to a sample of 180 participants with moderate, severe VI, and blindness, divided into three groups of 60 participants each:

- Control group: Participants with normal cognition.
- Case group: This group was further subdivided into two subgroups:
  - ◦ The MCI group: participants with MCI
  - ◦ The Dementia group: participants with mild to moderate dementia

## Materials

### Neuropsychological testing

A Diagnostic and Statistical Manual of Mental Disorders (DSM-5): The DSM5 criteria were applied to diagnose dementia and MCI [18]. It is a standardized criterion for classifying dementia and MCI based on clinical symptoms, cognitive decline, and functional impairment.
B Clinical Dementia Rating (CDR): The CDR scale assessed cognitive impairment severity and categorized participants according to dementia symptom severity. Participants were classified into the following categories [19]:
  0: Cognitively healthy
  0.5: MCI
  ≥1: Mild dementia
  ≥2: Moderate dementia
C. Clock Face Conceptualization (CFC) Subtest of the Verbal Clock Drawing Test (VCDT): This subtest was utilized to assess executive and visuospatial functioning. The CFC subtest consists of 10 questions with a 20-point total maximum score. It evaluates several aspects of clock drawing, including the shape of the clock face, the arrangement of digits, the number and size of hands, and the accurate placement of the hands at a specified time (11:10), with the following scoring system:
  2 points for a correct spontaneous answer
  1 point for a self-corrected or prompted answer
  0 points for an incorrect answer
D. ACE-III: The ACE-III is a comprehensive cognitive screening tool that assesses multiple cognitive domains, including attention, memory, fluency, language, and visuospatial abilities. The original ACE-III and the modified VIACE III were administered to all participants. The sensitivity, specificity, and cut-off points for identifying MCI and dementia were assessed using the VI-ACE III by comparing visually impaired participants' performance to those with normal cognition.

### Functional assessment

Functional status was evaluated using the Activities of Daily Living (ADL) [20] and Instrumental Activities of Daily Living (IADL) [21] scales. These measures indicate intact function in participants with normal cognition and MCI as well as impaired function in participants with dementia.

### Visual assessment

Visual acuity was measured using the Landolt Broken Rings chart [22] to assess the best binocular visual presentation. Both the original and modified versions of the ACE-III were administered at a near distance (test distance of 40 cm), with participants wearing their reading or corrective glasses. Standard illumination levels between 807 lx (lx) and 1345 lx (lx) were maintained during testing [23].

## Statistical analysis

The collected data were coded, tabulated, and statistically analyzed using IBM SPSS statistics (Statistical Package for Social Sciences) software version 28.0, IBM Corp., Chicago, USA, 2021. The normality of quantitative data was assessed using the Kolmogorov–Smirnov test. The data were described as mean ± SD (standard deviation) and compared using ANOVA and paired t-tests for original and modified items. Qualitative data was described as numbers and percentages and then compared using the Chi-square test and Fisher's Exact test. ROC curve analysis was conducted to determine the AUC, which assessed the performance and characteristics of various tests in accurately classifying participants with and without MCI or dementia. The Bonferroni test was used for post hoc comparisons.

The level of statistical significance was set at *p*-value ≤ 0.050. The internal consistency reliability of the VI- ACE-III was validated using Cronbach's alpha correlation coefficient.

Diagnostic characteristics were calculated as follows:

- Sensitivity = (True positive test / Total positive golden) × 100
- Specificity = (True negative test / Total negative golden) × 100
- Youden’s index = sensitivity + specificity – 1

## Results

The three groups were matched for the demographic variables, including age, gender, and education, as well as for the causes and grades of vision impairment, as with no statistically significant differences were found between the groups (Table 1).

**Table 1:** Demographic characteristics of the participants

Variables			Dementia Total = 60	MCI Total = 60	Control Total = 60	*p*-value
**Age (years)**		**60 − 69**	**33 (55.0%)**	**35 (58.3%)**	**34 (56.7%)**	**0.977**^**#**^
		**70 − 79**	19 (31.7%)	19 (31.7%)	18 (30.0%)	
		**80 − 89**	8 (13.3%)	6 (10.0%)	8 (13.3%)	
**Sex**		**Male**	28 (46.7%)	28 (46.7%)	31 (51.7%)	0.819^**#**^
		**Female**	32 (53.3%)	32 (53.3%)	29 (48.3%)	
**Education**		**Below university**	40 (66.7%)	36 (60.0%)	29 (48.3%)	0.119^**#**^
		**University**	20 (33.3%)	24 (40.0%)	31 (51.7%)	
**Degree of VI**		**Moderate**	15 (25.0%)	19 (31.7%)	18 (30.0%)	0.946^**#**^
		**Severe**	24 (40.0%)	21 (55.0%)	22 (36.7%)	
		**Blind**	21 (55.0%)	20 (33.3%)	20 (33.3%)	
**Causes of VI**	**Errors of refraction**		42 (70.0%)	48 (80.0%)	38 (63.3%)	0.128^**#**^
	**Cataract**		31 (51.7%)	29 (48.3%)	33 (55.0%)	0.766^**#**^
	**Glaucoma**		3 (5.0%)	5 (8.3%)	3 (5.0%)	0.793^**§**^
	**Age related macular degeneration**		14 (23.3%)	14 (23.3%)	16 (26.7%)	0.887^**#**^
	**Diabetic retinopathy**		14 (23.3%)	15 (25.0%)	22 (36.7%)	0.210^**#**^
	**Trauma**		3 (5.0%)	4 (6.7%)	6 (10.0%)	0.676^**§**^

(%) Data presented as number # Chi square test

^§^Fisher’s Exact test

*Abbreviation*: *MCI* mild cognitive impairment, *VI* Vision impairment

The dementia group exhibited the lowest mean scores for the ACE-III and VIACE-III, recorded at (45.4 ± 11.7 and 67.5 ± 11.2, respectively). The MCI group recorded scores of (56.4 ± 11.9 and 87.3 ± 8.4). The control group demonstrated the highest scores, recorded at (71.5 ± 11.7 and 101.8 ± 5.2, respectively). All study groups exhibited significant differences (*p*-value < 0.001), except for the original language test scores. There were significant differences between the dementia group (10.8 ± 5.2) and other groups. However, no significant difference was found between the MCI and control groups. In the original visuospatial tests, significant differences were noted only between the control group (8.3 ± 4.7) and the other groups, while no significant difference was found between the dementia and MCI groups Mean scores of the ACE-III and VI- ACE-III were lowest in dementia group (45.4 ± 11.7) (67.5 ± 11.2), followed by the MCI group (56.4 ± 11.9) (87.3 ± 8.4) and highest in the control group (71.5 ± 11.7) (101.8 ± 5.2), respectively. The differences were significant (*p*-value < 0.001) between all the study groups except for the scores of original language test (the differences were significant only between dementia group (10.8 ± 5.2) and other groups, with no significant difference between MCI and control groups) and the original visuospatial tests (where the differences were significant only between control group (8.3 ± 4.7) and other groups, with no significant difference between dementia and MCI groups (Table 2).

**Table 2:** Scores of both test items between the studied groups

Variables	Dementia Total = 60	MCI Total = 60	Control Total = 60	*p*-value
**Attention**	11.7 ± 1.7 ^**a**^	14.2 ± 1.1^**b**^	16.8 ± 1.3^**c**^	^ < 0.001*
**Memory**	16.9 ± 3.4^**a**^	20.3 ± 1.5^**b**^	24.1 ± 1.3^**c**^	^ < 0.001*
**Language (O)**	10.8 ± 5.2^**a**^	13.7 ± 5.8^**b**^	15.9 ± 6.0^**b**^	^ < 0.001*
**Language (M)**	19.0 ± 3.3^**a**^	23.3 ± 1.2^**b**^	24.7 ± 1.3^**c**^	^ < 0.001*
**CDT**	0.9 ± 0.9^**a**^	1.7 ± 1.2^**b**^	3.0 ± 1.6^**c**^	^ < 0.001*
**VCDT**	10.5 ± 2.0^**a**^	18.0 ± 1.4^**b**^	19.8 ± 0.4^**c**^	^ < 0.001*
**Visuospatial (O)**	4.4 ± 3.0^**a**^	5.7 ± 3.9^**a**^	8.3 ± 4.7^**b**^	^ < 0.001*
**Visuospatial (M)**	18.3 ± 3.7^**a**^	26.9 ± 2.0^**b**^	29.7 ± 1.2^**c**^	^ < 0.001*
**ACE-III**	45.4 ± 11.7^**a**^	56.4 ± 11.9^**b**^	71.5 ± 11.7^**c**^	^ < 0.001*
**VI- ACE-III**	67.5 ± 11.2^**a**^	87.3 ± 8.4^**b**^	101.8 ± 5.2^**c**^	^ < 0.001*

**^**ANOVA: Analysis of Variance test was used to compare more than two groups

^< 0.001*: *p*-value indicating statistical significance (*p* < 0.001). a, b, c: Used to represent groups identified as statistically similar or different after post hoc testing (Bonferroni correction)

*Abbreviations*: *O* Original, *M* Modified, *CDT* Clock Drawing Test, *VCDT* Verbal Clock Drawing Test, *MCI* mild cognitive impairment, *ACE-III* Addenbrooke’s Cognitive Examination III, *VI-ACEIII* vision impairment—Addenbrooke’s Cognitive Examination III

**Fig. 2:**
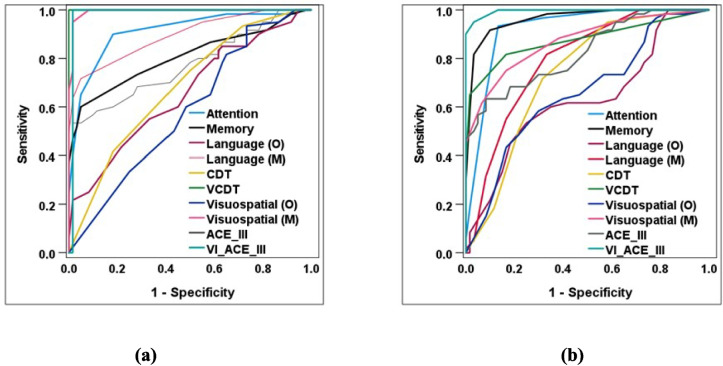
(ROC) curve for scores of ACE-III and VI- ACE-III in differentiating the dementia group from the MCI group (**a**) and MCI group from the control group (**b**) among all vision impairment grades

Using The ROC curve (Fig. 2 (a)) indicates that, the optimal cut-off point for dementia on the VI- ACE-III total score was calculated to be 84 out of 115, with a high diagnostic performance and characteristics (sensitivity of 100.0%sensitivity, specificity of 98.3% specificity, an Area under the curve (AUC) was of 0.983, and Youden’s Index (YI) of 98.3%). The VCDT exhibited excellent diagnostic performance Additionally, the VCDT demonstrated perfect diagnostic performance and characteristics in differentiating the dementia group from the MCI group, with optimal sub-score cut-off point 15/20 (sensitivity of 100.0% sensitivity, specificity of 100.0 specificity, an AUC was of 1.000, and YI of 100.0%), followed by attention with optimal sub-score cut-off point 14/18 (sensitivity of 90.0%sensitivity, specificity of 81.7%specificity, an AUC was of 0.911, and YI of 71.7%), and finally modified visuospatial with optimal sub-score cut-off point 24/31 (sensitivity of 95.0%sensitivity, specificity of 98.3% specificity, an AUC was of 0.993, and YI of 93.3%).

Using The ROC curve depicted in ROC curve in (Fig. 2 (b)) demonstrates that, VI- ACE-III had exhibited the highest diagnostic performance and characteristics in differentiating the MCI group from the control group, with an optimal cut-off point 94/115 (sensitivity of 95.0%sensitivity, specificity of 96.7%specificity, an AUC was of 0.995, and YI of 91.7%), followed by attention with optimal subscore cut-off point 16/18 (sensitivity of 93.3%sensitivity, specificity of 86.7%specificity, AUC was of 0.922, and YI of 80.0%), and finally memory tests, with optimal sub-score cut-off point 23/26 (sensitivity of 91.7%sensitivity, specificity of 90.0%specificity, an AUC was of 0.963, and YI of 81.7%).

In (Table 3), indicates that the performance speed of ACE-III and VI- ACE-III was the longest in the dementia group, followed by the MCI group, with the control group exhibiting the shortest performance speed. Significant differences were observed among all study groups. The performance speed of VI-ACE-III, in terms of total score and modified domains, was significantly shorter than that of the original versions.

**Table 3:** Performance speed of both test items and total score (minutes) between the studied groups

Scores	Dementia (Total = 60)	MCI (Total = 60)	Control (Total = 60)	*p*-value
**Attention**	3.6 ± 0.3	3.1 ± 0.1	2.5 ± 0.3	^ < 0.001*
**Memory**	2.4 ± 0.2	2.1 ± 0.1	1.8 ± 0.1	^ < 0.001*
**Language (O)**	3.9 ± 0.2	3.5 ± 0.1	3.0 ± 0.2	^ < 0.001*
**Language (M)**	2.8 ± 0.2	2.5 ± 0.1	2.0 ± 0.2	^ < 0.001*
***p*- value (language O vs. M)**	⌂ < 0.001*	⌂ < 0.001*	⌂ < 0.001*	
**CDT**	2.7 ± 0.2	2.3 ± 0.1	1.9 ± 0.2	^ < 0.001*
**VCDT**	1.2 ± 0.1	0.9 ± 0.1	0.7 ± 0.2	^ < 0.001*
***p*- value (CDT O vs. VCDT)**	⌂ < 0.001*	⌂ < 0.001*	⌂ < 0.001*	
**Visuospatial (O)**	4.1 ± 0.3	3.4 ± 0.3	2.5 ± 0.5	^ < 0.001*
**Visuospatial (M)**	3.4 ± 0.3	2.8 ± 0.2	2.2 ± 0.3	^ < 0.001*
***p*- value (visuospatial O vs. M)**	⌂ < 0.001*	⌂ < 0.001*	⌂ < 0.001*	
**ACE_III**	16.7 ± 1.2	14.3 ± 0.7	11.6 ± 1.2	^ < 0.001*
**VI_ACE_III**	13.4 ± 1.1	11.3 ± 0.6	9.2 ± 1.0	^ < 0.001*
***p*- value (ACE_III vs. VI_ACE_III)**	⌂ < 0.001*	⌂ < 0.001*	⌂ < 0.001*	

^ANOVA: Analysis of Variance test was used to compare more than two groups

⌂: A paired t-test was used to compare two related groups

^ < 0.001* or ⌂ < 0.001*: *p*-value indicating statistical significance (*p* < 0.001)

*Abbreviations*: *O* Original, *M* Modified, *CDT* Clock Drawing Test, *VCDT* Verbal Clock Drawing Test, *ACE-III* Addenbrooke’s Cognitive Examination III, *VI-ACE-III* vision impairment—Addenbrooke’s Cognitive Examination III

The optimal cut-off points and psychometric properties of the VI- ACE-III for detecting MCI and Dementia across various grades of vision impairment compared to the original ACE-III were addressed in (Table 4). VI- ACE-III had perfect diagnostic performance and characteristics in detecting dementia, with an optimal cut-off 84/115 (100.0% sensitivity, 100.0% specificity, and YI 100.0%) for both the moderate VI group and the Blind group. The optimal cut-off point for the severe VI group was 82/115 (95.8% sensitivity, 95.2% specificity, and YI 91.1%).

**Table 4:** Sensitivity, specificity and cutoff points for the original and modified test

Variables	Dementia & MCI		MCI & Control	
	ACE-III	VI- ACE-III	ACE-III	VI- ACE-III
**Moderate VI**				
** AUC**	0.796	1.000	0.895	0.997
***P*-Value**	0.003*	< 0.001*	< 0.001*	< 0.001*
** 95% CI**	0.646–0.947	1.000–1.000	0.788–1.000	0.988–1.000
** Cut point**	≤ 59	≤ 84	≤ 75	≤ 94
** Sensitivity**	53.3%	100.0%	84.2%	100.0%
** Specificity**	94.7%	100.0%	83.3%	94.4%
** Youden’s Index**	48.1%	100.0%	67.5%	94.4%
**Severe VI**				
** AUC**	0.809	0.952	0.919	0.995
***P*-Value**	< 0.001*	< 0.001*	< 0.001*	< 0.001*
** 95% CI**	0.674–0.944	0.861–1.000	0.828–1.000	0.982–1.000
** Cut point**	≤ 44	≤ 82	≤ 58	≤ 94
** Sensitivity**	66.7%	95.8%	85.7%	95.2%
** Specificity**	95.2%	95.2%	100.0%	95.5%
** Youden’s Index**	61.9%	91.1%	85.7%	90.7%
**BLIND**				
** AUC**	0.899	1.000	0.968	0.995
***P*-Value**	< 0.001*	< 0.001*	< 0.001*	< 0.001*
** 95% CI**	0.805–0.992	1.000–1.000	0.922–1.000	0.922–1.000
** Cut point**	≤ 44	≤ 84	≤ 56	≤ 95
** Sensitivity**	71.4%	100.0%	95.0%	100.0%
** Specificity**	100.0%	100.0%	85.0%	90.0%
** Youden’s Index**	71.4%	100.0%	80.0%	90.0%

^< 0.001*: *p*-value indicating statistical significance (*p* < 0.001)

*Abbreviations*: *MCI* mild cognitive impairment, *VI* vision impairment, *AUC* Area under the curve, *CI* Confidence interval, *ACE-III* Addenbrooke’s Cognitive Examination III, *VI-ACE-III* vision impairment—Addenbrooke’s Cognitive Examination III

It also demonstrated a high diagnostic performance and characteristics in detecting identifying MCI, with an optimal cut-off point of 94/115 for both moderate and severe VI and as well as 95/115 for the blind group (94.4% sensitivity and &specificity, YI 94.4% for moderate VI, 95.2% sensitivity, 95.5% specificity, YI 90.7% for the severe VI and as well as 100.0% sensitivity, 90.0%% specificity, and YI 90.0% for the blind group).

### Reliability

The study demonstrated strong internal consistency reliability in the dementia and MCI groups, as indicated by Cronbach's alpha values Our study showed good internal consistency reliability within the dementia and MCI group using Cronbach’s alpha values (α = 0.866, α = 0.771), respectively.

## Discussion

Recent longitudinal studies have demonstrated significant associations between vision loss and accelerated cognitive decline, along with an elevated risk of dementia [24]. The 2024 update in *Lancet Dementia Prevention* identifies vision loss as one of the 14 modifiable risk factors for dementia [25]. Standard cognitive tests often depend on intact sensory functions, making it difficult to assess cognitive abilities accurately in individuals with sensory impairments.

Consequently, there is a pressing need for a cognitive screening test specifically adapted for elderly individuals with visual impairments [26]. Previous attempts to adapt cognitive assessments for individuals with visual impairments have been hindered by considerable limitations. These constraints primarily stem from the insufficiency of current psychometric tools and the complexities associated with ensuring accessibility, particularly for individuals with concomitant sensory impairments, such as hearing loss [27]. Furthermore, the reliance on auditory and tactile modalities may inadequately capture the full extent of cognitive decline, especially in individuals who have developed compensatory mechanisms to mitigate sensory impairments [28]. Modifying scoring techniques, individual test items, or adopting non-standardized administration methods poses a potential threat to the integrity of the cognitive assessment [29]. Many assessment tools fail to account for the unique cognitive profiles of individuals with visual impairments, and the testing environment can significantly impact performance. Variability in assessors' training and the lack of comprehensive support systems may further increase anxiety and hinder accurate performance [30].

Examples of cognitive assessment tools specifically developed for elderly populations with visual impairments, include the Hong Kong Montreal Cognitive Assessment (HK-MoCA) and Vision Cog. These tools effectively address the limitations inherent in traditional neuropsychological assessments that rely on visual stimuli. The HK-MoCA represents a culturally and sensorially adapted version of the original MoCA, designed to minimize dependence on visual tasks by emphasizing verbal recall, orientation, and language proficiency. This instrument has been validated for application among older adults with visual deficits in Hong Kong [31].

Vision Cog is explicitly designed for individuals with visual impairments, employing auditory and tactile modalities to evaluate memory, attention, and executive function. It has demonstrated strong psychometric properties in multicenter trials [32].

This study was conducted to provide a validated version of ACE-III adapted for visually impaired elderly individuals. This study represents the first research conducted on Arabic speakers. In this study, we employed a method of item substitution in the development of VI-ACE-III, choosing to use verbal and primarily printed materials rather than removing items. This method was selected to improve the validity and reliability of the assessment tool. Studies demonstrate that larger print materials are crucial for promoting independence in visually impaired elderly individuals [33]. Research indicates that reading large print requires less cognitive effort compared to interpreting tactile information, particularly among older adults facing cognitive decline [34].

Incorporating verbal elements in cognitive assessments may alleviate the difficulties associated with visual impairments. Research published in the Journal of *Visual Impairment & Blindness* indicates that participants with vision impairment exhibited superior performance on cognitive tests that utilized verbal instructions and responses compared to those that depended solely on written text. The use of auditory stimuli enables individuals to leverage their intact auditory processing skills, thus enhancing performance on these assessments [35].

The findings indicated that the optimal cut-off point on the modified ACE-III for dementia was 84 out of 115, yielding 100% sensitivity and 98.3% specificity. The study determined the optimal cut-off point for dementia on the standard version of ACE-III to be 44 out of 100, with a sensitivity of 53.3% and a specificity of 98.3%. The findings differ from the Arabic ACE-III scores, which identified an optimal cut-off point of 72 out of 100, exhibiting 89% sensitivity and 95% specificity. The optimal cut-off point for the modified ACE-III in identifying MCI is 94 out of 115, achieving 95% sensitivity and 96.7% specificity. The ideal cut-off score for the standard version of ACE-III in MCI is 59 out of 100, resulting in 63.3% sensitivity and 91.7% specificity. The Arabic ACE-III scores reveal an optimal cut-off point of 81, demonstrating 75% sensitivity and 82% specificity.

The observed differences in scores among visually impaired individuals on cognitive tests indicate the impact of vision impairment on test results, aligning with previous research findings like the study that investigated the effects of visual impairment on the Montreal Cognitive Assessment (MoCA) where simulated reductions in visual acuity led to lower cognitive performance scores [36].

The VCDT demonstrates a higher specificity of 100% for diagnosing dementia compared to the total scores of the VI-ACE-III, with a specificity of 98.3%. This finding indicates that the VCDT may serve as an independent screening tool for dementia, especially among visually impaired elderly individuals. The VCDT demonstrates greater specificity and improved performance speed (1.2 ± 0.1 s) compared to the clock drawing test (2.7 ± 0.2 s).

Individuals with VI often experience significant fatigue over time as a result of cognitive overload due to the need to compensate for reduced visual input [37]. Consequently, using rapid screening tools like VCDT can be effective in the early identification of dementia among visually impaired elderly individuals.

### Strengths and limitations of the study

The strength of this study lies in its uniqueness as the first research focusing on Arabic-speaking visually impaired elderly individuals in Egypt. The performance speed on the modified test was compared to the original version across each cognitive domain. The study also compared the diagnostic performance and characteristics of the modified test among various grades of vision impairment. This area has garnered minimal research interest, as indicated in the Supplementary Tables.

However, our study has several limitations. First, cognitive status classification was based on DSM-5 criteria, CDR, and clinical neuropsychological assessment due to the lack of a valid cognitive test for the visually impaired in Egypt. Second, the generalizability of the current study’s findings may be constrained by the participants’ homogeneous educational attainment, as all individuals possessed a baseline level of formal education. Future research should prioritize the inclusion of illiterate and low-educated elderly individuals. Third, the visual acuity was measured by the Landolt broken ring chart, which is a less sophisticated screening measure compared to other methods, such as log MAR charts, which offer greater discriminant validity, sensitivity to inter-ocular differences, and reliability. Moreover, other visual functions potentially influencing cognitive test performance, including contrast sensitivity, visual field, and stereoscopic acuity, were not assessed. We incorporated methodological adaptations to address aspects of contrast sensitivity and visual field. Specifically, we utilized high-contrast print materials to optimize task legibility, thereby implicitly addressing contrast-related challenges that might confound cognitive task performance. Furthermore, to ensure broad spatial coverage of visual stimuli, items were projected in an H-shaped configuration, systematically spanning all quadrants of the visual field (superior, inferior, nasal, and temporal). This design aimed to mitigate biases from localized visual deficits and approximate a more holistic assessment of functional vision.

Future studies are needed to integrate contrast sensitivity and visual fields to provide a clearer picture of how different aspects of visual impairment influence cognitive performance.

## Conclusion

This study highlights the importance of developing and validating cognitive assessment tools specifically tailored for visually impaired elderly individuals. The VI-ACE-III demonstrates potential as a diagnostic instrument for dementia and MCI in this population, with significant implications for future research and clinical practice.

## Supplementary Information

Supplementary Material 1.

Supplementary Material 2.

## Data Availability

All data generated or analyzed during this study are included in this published article.

## Abbreviations

ACE-III: Addenbrooke’s Cognitive Examination III
VI-ACE-III: Vision impairment Addenbrooke’s Cognitive Examination III
ADL: Activities of Daily Living
AUC: Area Under the Curve
ROC: Receiver Operating Characteristic curve
CDR: Clinical Dementia rating scale
MCI: Mild Cognitive Impairment
VCDT: Verbal clock drawing test
logMAR: Log of minimum angle of resolution
WHO: World health organization
GDS: Geriatric depression scale
CSDD: Cornell scale for depression
DSM–5: Diagnostic and Statistical Manual of Mental Disorders
CDR: Clinical Dementia Rating
IADL: Instrumental activities of daily living
YI: Youden's index
cfc: The clock face conceptualization
MOCA: Montreal Cognitive Assessment
HK-MoCA: Hong Kong Montreal Cognitive Assessment
