# Supplementary material for: Adaptation and validation of the modified Egyptian Arabic version of Addenbrooke’s Cognitive Examination III (VI-ACE-III) for assessing cognitive impairment in visually impaired elderly
Source: BMC Geriatr. 2025 Mar 4;25:145. doi: 10.1186/s12877-025-05784-1 (PMC11877952; doi:10.1186/s12877-025-05784-1)
Supplement: Supplementary file 1 — Supplementary Material 1. [file 12877_2025_5784_MOESM1_ESM.docx]

**Supplementary table 1**

Diagnostic performance among different grades of vision impairment for both the original and modified test items in differentiating dementia from the MCI group

| **Variables** | **AUC** | **p-value** | **95% CI** | **Cut point** | **Sensitivity** | **Specificity** | **Youden’s Index** |
| --- | --- | --- | --- | --- | --- | --- | --- |
| **Moderate vision impairment** | | | | | | | |
| **Attention** | 0.791 | 0.004* | 0.626–0.957 | ≤13 | 53.3% | 94.7% | 48.1% |
| **Memory** | 0.668 | 0.096 | 0.475–0.862 | ≤20 | 60.0% | 68.4% | 28.4% |
| **Language (O)** | 0.667 | 0.099 | 0.471–0.862 | ≤17 | 46.7% | 94.7% | 41.4% |
| **Language (M)** | 0.840 | 0.001* | 0.706–0.974 | ≤22 | 60.0% | 94.7% | 54.7% |
| **CDT** | 0.735 | 0.020* | 0.567–0.903 | ≤3 | 73.3% | 63.2% | 36.5% |
| **VCDT** | 1.000 | <0.001* | 1.000–1.000 | ≤15 | 100.0% | 100.0% | 100.0% |
| **Visuospatial (O)** | 0.670 | 0.093 | 0.488–0.852 | ≤10 | 73.3% | 63.2% | 36.5% |
| **Visuospatial (M)** | 0.974 | <0.001* | 0.925–1.000 | ≤25 | 100.0% | 89.5% | 89.5% |
| **Severe vision impairment** | | | | | | | |
| **Attention** | 0.952 | <0.001* | 0.893–1.000 | ≤14 | 95.8% | 90.5% | 86.3% |
| **Memory** | 0.870 | <0.001* | 0.758–0.982 | ≤19 | 75.0% | 100.0% | 75.0% |
| **Language (O)** | 0.689 | 0.030* | 0.533–0.846 | ≤9 | 54.2% | 85.7% | 39.9% |
| **Language (M)** | 0.945 | <0.001* | 0.886–1.000 | ≤21 | 79.2% | 95.2% | 74.4% |
| **CDT** | 0.673 | 0.048* | 0.514–0.831 | ≤1 | 41.7% | 81.0% | 22.6% |
| **VCDT** | 1.000 | <0.001* | 1.000–1.000 | ≤15 | 100.0% | 100.0% | 100.0% |
| **Visuospatial (O)** | 0.564 | 0.460 | 0.394–0.735 | ≤8 | 95.8% | 23.8% | 19.6% |
| **Visuospatial (M)** | 0.999 | <0.001* | 0.995–1.000 | ≤23 | 95.8% | 100.0% | 95.8% |
| **blind vision** | | | | | | | |
| **Attention** | 0.954 | <0.001* | 0.895–1.000 | ≤14 | 85.7% | 95.0% | 80.7% |
| **Memory** | 0.833 | <0.001* | 0.712–0.955 | ≤18 | 61.9% | 90.0% | 51.9% |
| **Language (O)** | 0.606 | 0.246 | 0.432–0.780 | ≤7 | 23.8% | 95.0% | 18.8% |
| **Language (M)** | 0.868 | <0.001* | 0.752–0.984 | ≤22 | 66.7% | 100.0% | 66.7% |
| **CDT** | 0.692 | 0.036* | 0.528–0.855 | ≤1 | 66.7% | 65.0% | 31.7% |
| **VCDT** | 1.000 | <0.001* | 1.000–1.000 | ≤15 | 100.0% | 100.0% | 100.0% |
| **Visuospatial (O)** | 0.533 | 0.715 | 0.352–0.714 | ≤5 | 100.0% | 15.0% | 15.0% |
| **Visuospatial (M)** | 1.000 | <0.001* | 1.000–1.000 | ≤24 | 100.0% | 100.0% | 100.0% |

Abbreviations: (O) Original; (M) Modified; CDT (Clock Drawing Test); VCDT (Verbal Clock Drawing Test); ^AUC: Area under curve. *Significant. CI: Confidence interval.
