# Supplementary material for: Adaptation and validation of the modified Egyptian Arabic version of Addenbrooke’s Cognitive Examination III (VI-ACE-III) for assessing cognitive impairment in visually impaired elderly
Source: BMC Geriatr. 2025 Mar 4;25:145. doi: 10.1186/s12877-025-05784-1 (PMC11877952; doi:10.1186/s12877-025-05784-1)
Supplement: Supplementary file 2 — Supplementary Material 2. [file 12877_2025_5784_MOESM2_ESM.docx]

**Supplementary table 2**

Diagnostic performance among different grades of vision impairment for both the original and modified test items in differentiating MCI from the Control group

| **Variables** | **AUC** | **p-value** | **95% CI** | **Cut point** | **Sensitivity** | **Specificity** | **Youden’s Index** |
| --- | --- | --- | --- | --- | --- | --- | --- |
| **moderate vision impairment** | | | | | | | |
| **Attention** | 0.896 | <0.001* | 0.787–1.000 | ≤16 | 100.0% | 77.8% | 77.8% |
| **Memory** | 0.955 | <0.001* | 0.891–1.000 | ≤22 | 78.9% | 100.0% | 78.9% |
| **Language (O)** | 0.746 | 0.011* | 0.582–0.909 | ≤24 | 89.5% | 55.6% | 45.0% |
| **Language (M)** | 0.906 | <0.001* | 0.814–0.999 | ≤25 | 84.2% | 83.3% | 67.5% |
| **CDT** | 0.880 | <0.001* | 0.760–1.000 | ≤4 | 84.2% | 88.9% | 73.1% |
| **VCDT** | 0.807 | 0.001* | 0.662–0.952 | ≤20 | 68.4% | 88.9% | 57.3% |
| **Visuospatial (O)** | 0.734 | 0.015* | 0.563–0.905 | ≤13 | 89.5% | 61.1% | 50.6% |
| **Visuospatial (M)** | 0.788 | 0.003* | 0.640–0.936 | ≤30 | 68.4% | 83.3% | 51.8% |
| **severe vision impairment** | | | | | | | |
| **Attention** | 0.940 | <0.001* | 0.868–1.000 | ≤16 | 100.0% | 81.8% | 81.8% |
| **Memory** | 0.957 | <0.001* | 0.897–1.000 | ≤22 | 90.5% | 90.9% | 81.4% |
| **Language (O)** | 0.765 | 0.003* | 0.610–0.920 | ≤11 | 57.1% | 95.5% | 52.6% |
| **Language (M)** | 0.741 | 0.007* | 0.594–0.888 | ≤25 | 76.2% | 59.1% | 35.3% |
| **CDT** | 0.895 | <0.001* | 0.800–0.990 | ≤3 | 81.0% | 86.4% | 67.3% |
| **VCDT** | 0.947 | <0.001* | 0.877–1.000 | ≤20 | 95.2% | 81.8% | 77.1% |
| **Visuospatial (O)** | 0.788 | 0.001* | 0.641–0.935 | ≤6 | 71.4% | 81.8% | 53.2% |
| **Visuospatial (M)** | 0.971 | <0.001* | 0.928–1.000 | ≤29 | 95.2% | 90.9% | 86.1% |
| **blind vision** | | | | | | | |
| **Attention** | 0.960 | <0.001* | 0.906–1.000 | ≤16 | 80.0% | 100.0% | 80.0% |
| **Memory** | 0.993 | <0.001* | 0.976–1.000 | ≤23 | 90.0% | 100.0% | 90.0% |
| **Language (O)** | 0.703 | 0.028* | 0.539–0.866 | ≤12 | 90.0% | 50.0% | 40.0% |
| **Language (M)** | 0.729 | 0.013* | 0.561–0.896 | ≤25 | 85.0% | 60.0% | 45.0% |
| **CDT** | 0.583 | 0.372 | 0.402–0.763 | ≤2 | 80.0% | 40.0% | 20.0% |
| **VCDT** | 0.875 | <0.001* | 0.755–0.995 | ≤19 | 75.0% | 100.0% | 75.0% |
| **Visuospatial (O)** | 0.656 | 0.091 | 0.484–0.829 | ≤4 | 80.0% | 50.0% | 30.0% |
| **Visuospatial (M)** | 0.895 | <0.001* | 0.800–0.990 | ≤29 | 85.0% | 75.0% | 60.0% |

Abbreviations: (O) Original; (M) Modified; CDT (Clock Drawing Test); VCDT (Verbal Clock Drawing Test); ^AUC: Area under curve. *Significant. CI: Confidence interval.
